# The clinical value of brain computerised tomography in a general hospital psychiatric service

**DOI:** 10.4102/sajpsychiatry.v23i0.1050

**Published:** 2017-06-01

**Authors:** Usha Chhagan, Jonathan K. Burns

**Affiliations:** 1 Department of Psychiatry, Nelson R. Mandela School of Medicine, University of KwaZulu-Natal, South Africa

## Abstract

**Background:**

The use of neuroimaging modalities in psychiatry has been evaluated in several studies. The vast majority seem to suggest that neuroimaging may be overutilised in psychiatry. There is a significant constraint on availability and cost related to neuroimaging of patients at general state medical facilities. The routine use of computerised tomography (CT) scanning is thus questioned.

**Methods:**

A retrospective analysis was undertaken of all psychiatric inpatients who had CT scans performed from 01 January 2011 to 31 December 2012. Demographic data, mental state examination, physical examination findings, substance use and diagnosis upon admission were recorded. The relationship between these variables and CT scan findings was analysed.

**Results:**

A total of 897 admissions were retrospectively analysed. One hundred and three patients had documented CT scan imaging. In total, 17 of the 23 patients with abnormal findings on CT scan were found to be psychotic (74.0%). The remaining 26.0% included depression and dementia. There was no statistically significant difference between the normal and abnormal CT scan groups with regard to gender, age, family history, substance use and physical examination. The majority (65.2%) had cerebral atrophy and/or cerebral calcifications. A smaller group of other documented findings was noted.

**Conclusions:**

Selective indications and clinical markers may be utilised in order to justify brain imaging studies rather than performing them routinely. That being true, a multicentre study with a larger sample size is indicated to further improve the statistical significance and assist in formulating a more concrete guideline for neuroimaging of psychiatric patients.

## Introduction

When evaluating a patient, a psychiatrist is often faced by the following dilemma regarding neuroimaging: either that no further imaging is warranted or to request further neuroimaging by computerised tomography (CT) scan or magnetic resonance imaging (MRI) scan. The selection criteria for CT scanning eligibility vary among psychiatric units. Published data have also showed varied results at different units. The purpose of this study was to describe the current trends in neuroimaging of mentally ill patients as well as to demonstrate the value of the CT scan as it is currently utilised. The study protocol entailed determining the number of patients imaged by CT scanning and documenting the psychiatric diagnoses made. Key objectives further included identification of the number of patients with abnormal findings on CT scanning and to assess if the CT findings impacted the treatment administered.

In 1992, in a retrospective study, Berk analysed the indications for CT scanning in psychiatric inpatients and concluded that there are specific clinical variables that correlated with scan abnormality. Furthermore these variables, including presence of neurological abnormality, diagnosis of delirium or dementia, organic mental state abnormality and EEG abnormality could be used as clinical guidelines for referral for CT scan.^1^ Other relevant clinical factors to consider are older age of patient, history of prior head injury, alcohol abuse and neuropsychological test abnormality.

Emsley et al., in a retrospective study of 100 patients referred for CT scans, reported abnormalities in 61 patients. Of these patients, 23% had focal brain lesions. The conclusion of the study was that certain clinical factors are useful in identifying patients that may warrant CT scanning.^2^ This study also highlighted the fact that a too restrictive approach in selection for CT scanning may risk the failure to detect a significant intracranial lesion.

Computerised tomography scanning may be of benefit in psychiatric practice in one of two ways. In the first instance, CT scanning can serve to confirm or exclude the presence of an organic cause for the clinically identified neurological abnormality. On the contrary, some recommend, as an equivalent to baseline blood investigations, the routine use of CT scanning as a screening tool in all psychiatric patients.^3^

Larson et al. in a retrospective review of 123 patients provided an alternative to this latter approach. Of the patients in the study group, 85% were classified as having normal neuroimaging or normal except for cerebral atrophy. All the patients who showed abnormalities on CT scanning manifested with true positive findings on clinical examination. They, therefore, recommended a concept of using a ‘rule in’ approach advocating the use of CT scanning in those patients who presented with significant focal findings on neurological examination rather than a general ‘rule out’ approach.^4^ This method allows for greater cost effectiveness whilst still maintaining a high level of selectivity and specificity – an approach that would have great benefit to a constrained health care system. Furthermore, according to the authors, over-reliance on CT may lead to failure to diagnose many other treatable medical illnesses that can present as psychiatric illnesses, with potentially hazardous results.

National Institute for Health and Care Excellence (NICE) Guidelines^5^ of 2008 recommended that neuroimaging is not to be a routine part of the initial investigations for the management of first-episode psychosis patients. The appraisal committee reviewed evidence supplied by several sources including submissions by patient care groups, manufacturers of CT equipment, professional or specialist groups and commentator organisations. The assessment group concluded that it was not cost-effective to routinely image such patients by CT scanning as this modality resulted in positive findings that would influence clinical management in only 0.5% of people with psychosis (range of 0% – 5%).

## Methods

The study was designed as a retrospective analysis of all admissions to the psychiatry unit at Addington Hospital, Durban, from 01 January 2011 to 31 December 2012 who had a CT brain scan performed. The 103 patients that were imaged by CT brain scanning were closely analysed. The age, gender, past psychiatric history, significant family history, substance use, medical history, physical and mental state examination findings, admission Diagnostic and Statistical Manual (DSM) IV diagnosis and treatment administered were recorded for each of the patients undergoing CT scanning.

The CT scans were performed in the radiology department using a General Electric (GE) multislice CT scanner. The radiologist on duty individually reported all scans. The findings of each CT scan performed were recorded and correlated with the above-mentioned clinical factors. The findings of these reports were collated. The CT findings were correlated with the patient demographics, psychiatric diagnosis and clinical evaluation.

## Data analysis

Data were analysed using IBM SPSS (version 21.0). Descriptive statistics were calculated for demographic variables as well as clinical criteria and radiological findings.

## Results

Of a total of 897 admissions in the 2-year study period, 103 patients had documented CT scan imaging. These 103 patients represented 11.5% of all adult admissions to the psychiatric unit. The age range included patients between 18 and 85 years. Of these, 45 were men and 58 were women. Of the 103 patients who underwent CT scans, 23 (22%) had documented imaging abnormalities. These imaging findings have been enumerated and correlated with demographic details as well as clinical diagnosis in each patient (Table 1).

**TABLE 1:** Correlations of demographic and clinical factors with normal versus abnormal computerised tomography scan results.

Characteristics	Total (%)	Normal CT (%)	Abnormal CT (%)	Chi-square	*P*	Odds ratio (95% CI)
**Gender**						
Male	45 (43.7)	34 (76)	11 (24)	0.21	0.65	0.81
Female	58 (56.3)	46 (79)	12 (21)			(0.32–2.05)
**Age**						
18–30	41 (39.8)	32 (78)	9 (22)	5.72	0.25	
31–40	23 (22.3)	21 (91)	2 (9)			
41–50	15 (14.6)	12 (80)	3 (20)			
51–60	11 (10.7)	7 (64)	4 (36)			
➢ 60	13 (12.6)	8 (62)	5 (38)			
**Family history**						
No	88 (85.4)	67 (76)	21 (24)	0.82	0.37	0.49
Yes	15 (14.6)	13 (87)	2 (13)			(0.10–2.35)
**Substance use**						
No	56 (54.4)	43 (77)	13 (23)	0.06	0.81	0.89
Yes	47 (45.6)	37 (79)	10 (21)			(0.35–2.30)
**Physical exam abnormal**						
No	85 (82.5)	67 (79)	18 (21)	0.37	0.54	1.43
Yes	18 (17.5)	13 (72)	5 (28)			(0.45–4.55)
**Psychotic disorder**						
No	11 (10.7)	5 (45)	6 (55)	6.0	0.01*	0.19
Yes	92 (89.3)	75 (82)	17 (18)			(0.05–0.69)
**Depressive disorder**						
No	85 (82.5)	65 (76)	20 (24)	0.02	0.53	0.65
Yes	18 (17.5)	15 (83)	3 (17)			(0.17–2.48)
**Cognitive disorder**						
No	96 (93.2)	76 (79)	20 (21)	1.83	0.18	2.85
Yes	7 (6.8)	4 (57)	3 (43)			(0.59–13.78)

* Significance set at *p* < 0.05

CI, confidence interval; CT, computerised tomography.

In total, 17 (74%) of the 23 patients with abnormal findings on CT scanning were found to be psychotic. The remaining 6 (26%) included depression and dementia. There was no statistically significant difference between the normal and abnormal CT scan groups with regard to gender, age, family history, substance use and physical examination. Notably, of the psychiatric diagnoses that we found in the study, psychotic disorders showed an inverse relationship to abnormality on CT scan. Of the 92 patients with psychosis, 75 (82%) had normal CT scans (*p* = 0.01). In the group of patients with depression, a similar trend is noted with 15 (83%) of the 18 patients demonstrating normal CT scans; however, these were not statistically significant. Three (43%) of the seven patients with a diagnosis of a cognitive disorder were found to have abnormalities on CT scanning. Because of the small sample size, this failed to show statistical significance.

Of the 23 patients with positive scan findings, the majority (65.2%) had atrophy of the cerebral hemispheres and/or cerebral calcifications, which were either focal granulomas or basal ganglia calcifications (15/23). A smaller group of other documented findings was noted (Table 2).

**TABLE 2:** Distribution of findings documented for computerised tomography scans.

*N* = 103	Atrophy	Calcification including calcified granuloma	Calcification and atrophy	Multiple infarcts + atrophy	Other†	Normal CT
Findings	7	7	1	1	7	80

† Other findings included: (1) Colloid cyst; (2) Communicating hydrocephalus; (3) Occipital arachnoid cyst; (4) Single left parietal hypodensity – suspected infarct; (5) Right basal ganglia lacunar infarct; (6) Old left basal ganglia infarct; (7) Mastoiditis.

CT, computerised tomography.

## Discussion

The clinical approach to a patient necessitates the evaluation for an underlying neurological disorder or other organic aetiology, which may impact further management. The identification of an underlying causal or otherwise clinically occult neurological illness by neuroimaging may result in quite a different treatment path, and it is for this reason that psychiatrists worldwide are faced with this dilemma of whom to image and whom not to. In the 1980s when CT scanning, and later MRI scanning, came into vogue, several recommendations were made following research publications of the usefulness of neuroimaging in the detection of underlying brain lesions or other disorders. In 1984, Weinberger^3^ formulated a list of indications for CT scanning. This was based on an identification of the structural brain diseases associated with several psychiatric conditions.

In our study, 22% of patients had abnormal CT scans. The majority of these were because of cerebral atrophy and calcifications, which did not influence diagnosis and future patient management. A smaller number (7/23) showed other findings. In this latter group, only one finding, namely that of communicating hydrocephalus, altered the treatment of the patient who was referred for further medical management. Thus, abnormalities on CT scanning did not impact the treatment administered for 22 of the 23 patients; however, the small sample size in our study contributes to the lack of statistically significant findings.

This study adds to and supports the previous findings by Agzarian et al.,^6^ Bain^7^ and McClellan et al.^8^ All three of these studies drew a similar conclusion that routine CT scanning of all patients may not be warranted. Agzarian et al.^6^ showed specific abnormalities on neuroimaging in only 5% of 397 cases. The study group concluded that there was a similar probability of finding an intracranial abnormality in neuroimaging of patients presenting with a variety of psychiatric illnesses when compared to the general population. Bain^7^ in 1998 described a less remarkable outcome for CT studies in first-episode psychotic patients. The conclusion was that further prospective studies were necessary to better define populations in which CT scanning is indicated. The study was, however, biased by the sample population being largely restricted to young military personnel who were otherwise healthy as well as insensitive neurological screenings. Further support for this recommendation that routine use of CT scanning was not indicated in psychiatry inpatients was provided by McClellan et al.^8^ During the 3-year study period, the authors demonstrated abnormalities that were thought to be clinically unrelated to the patients’ psychiatric conditions. On the basis of these findings, the authors concluded that there was no justification for routine CT scanning in patients admitted to the hospital for psychiatric disorders. They therefore did not support the routine imaging of psychiatric patients.

There is an ongoing concern regarding the potential cancer risk from ionising radiation, particularly in young patients undergoing CT scanning. Pearce et al.^9^ assessed the risk of leukaemia and brain tumour development in a large cohort of children and young adults over a long study period. They demonstrated that although the cumulative risk of radiation from brain CT scans is low, their use in young adults and in particular, children, should be kept to a minimum. Alternative imaging by MRI is recommended in younger patients who have neurological signs warranting imaging.

A number of other studies have shown that the routine use of CT imaging, particularly in first-episode psychosis,^10,11^ is of little benefit in younger patients^12^ and in the absence of focal neurological signs.^13^

In a South African study conducted by Jeenah and Moosa, on mentally ill inpatients, CT scanning detected a significant number of abnormalities. In contrast to the above studies, this study, conducted at Chris Hani Baragwanath Hospital, further concluded that clinical abnormalities may not be reliable predictors of abnormal CT scans.^14^

## Limitations

Methodological limitations included the retrospective nature of the study and the clinical assessment of each patient having been made by different clinicians attending patients in the unit. The decision to proceed with a CT scan is dependent on the clinical presentation as well as the clinician attending the patient. In addition, the reporting radiologists were not blinded to the clinical details as reporting was performed during the routine work day. The radiological reports were performed by different reporting radiologists. This is unavoidable in a retrospective analysis as the studies were already performed and reported in a department that has staff working on a rotational basis.

## Conclusion

Neuroimaging in psychiatric practice has progressed significantly, particularly so in the last decade. However, despite these advances, the routine use of CT scanning in psychiatry has not been shown to impact significantly the management of patients. It has been suggested that selected indications and clinical markers be utilised in order to justify brain imaging studies, rather than doing them routinely. The need for proper imaging guidelines is mandatory especially in the presence of stringent budgetary constraints. Currently, at most state units, CT scanning is the available modality with few major centres having access to MRI scanning as well. In developing countries, health care workers are faced with the challenges of increasing health care costs and limited resources. Added to this, in South Africa, with the ever-growing concern of litigation, clinicians are forced into practising defensive medicine and overinvestigate patients.

The American Psychiatric Association (APA) Practice Guideline for the psychiatric evaluation of adults states that the clinical use of neuroimaging for planning of patient-specific treatment has not yet been shown. Further research is required to define a clinical role for structural neuroimaging in diagnoses, monitoring disease progression and predicting prognoses.^15^ Nationally, there is a lack of well-defined guidelines to assist in the selection of psychiatric patients to be referred for imaging studies. Protocols need to be formulated to guide this screening process. That we are exposed to a variety of illnesses especially infective conditions, not frequently seen in developed countries, may also necessitate the establishment of suitable guidelines that may be locally applicable. A multicentre study with a larger sample size is indicated to further improve the statistical significance and assist in formulating a more practical guideline for neuroimaging of psychiatric patients. The role of neuroimaging in psychiatry may then be more clearly established.
